# Genetic and environmental determinants of dental fluorosis: a case‒control study of DLX3, ESR1, and ESR2 variants in a high-fluoride region of Mexico

**DOI:** 10.1186/s12903-025-07109-5

**Published:** 2025-10-31

**Authors:** Sergio Manuel Salas Pacheco, Luis Antonio Ortiz Lopez, Leslie Karina Gamboa Guerrero, Omar Alejandro Tremillo Maldonado, Ada Agustina Sandoval Carrillo, Edna Madai Mendez Hernandez, Osmel La llave Leon, Lilia Velez Velez, Francisco Xavier Castellanos Juarez, Luis Javier Solis Martinez, Marcelo Gomez Palacio Gastelum, Gamaliel Ortiz Sarabia, Jose Manuel Salas Pacheco

**Affiliations:** 1 Faculty of Dentistry, Juarez University of Durango State, Durango, México; 2 Institute for Scientific Research, Juarez University of Durango State, Durango, México

**Keywords:** Dental fluorosis 1, DLX3 2, ESR1 3, ESR2 4, F 5

## Abstract

Dental fluorosis (DF) is a condition caused by prolonged fluoride (F) exposure during tooth development, leading to enamel changes. Clinically, it presents as opaque white spots and, in severe cases, striations, mottling, or enamel loss. Although chronic fluoride exposure is the main factor, severity does not always align with fluoride levels, suggesting genetic influences, such as variants in the DLX3, ESR1 and ESR2 genes, which regulate tooth development. This cross-sectional case–control study included 79 women from Durango, Mexico. The severity of dental fluorosis was assessed across the entire mouth via the Thylstrup–Fejerskov Index (TFI), with the highest score recorded for evaluation. The classification followed the World Health Organization (WHO) guidelines from Geneva, 1997, categorizing severity into mild, moderate, and severe groups. The fluoride concentrations in the water and urine were quantified via potentiometry. Genotyping was conducted via real-time PCR. The average concentration of fluoride (ppm) was 5.38(± 2.73 SD) for tap water, 4.79±(4.60 SD) for urine and 2.32(± 2.78 SD) for drinking water TFI followed the next distribution: 72.1% of the participants had moderate, 22.7% had mild, and 5.2% had severe fluorosis. A positive correlation between F in drinking water and F in urine. (0.46, *p* = 0.001) and a negative correlation between F in drinking water and the mean corpuscular volume (MCV) (-0.41, *p* = 0.01) was found. Additionally, a significant association was identified between the recessive model of the rs2278163 DLX3 and rs12154178 ESR1 polymorphisms and mild and moderate fluorosis (*p* = 0.02, OR = 0.25, 95% CI: 0.07–0.88 and *p* = 0.034, OR = 0.31, 95% CI: 0.10–0.95, respectively).

## Background

 Dental fluorosis presents as an alteration in the development of dental enamel due to high and prolonged concentrations of fluoride during the development of teeth. This alteration results in enamel with a lower mineral content and higher porosity [1].

The manifestation of fluoride toxicity during tooth development is dependent on both the duration of exposure and the concentration of fluoride. Excessive fluoride levels during enamel formation result in metabolic disruptions within ameloblasts, leading to porosities in the developing enamel. These porosities impair the proper development and maturation of the enamel, ultimately causing structural defects [2]. Elevated fluoride concentrations reduce the availability of calcium ions, which in turn diminishes the proteolytic capacity of the enamel matrix [3]. Clinically, dental fluorosis is characterized by opaque white areas on the enamel, often accompanied by striations, mottling, or discontinuities in the enamel structure [4]. These clinical manifestations have been classified by numerous researchers and professionals. The Thylstrup and Fejerskov index (TFI) provides a comprehensive assessment of dental fluorosis, capturing early enamel changes that may not be detected by other indices [5]. Based on the severity of dental fluorosis as measured by the TFI, four groups have been proposed: sound enamel (TFI = 0), mild (TFI = 1–3), moderate (TFI = 4–6), and severe (TFI = 7–9) [6].

In the northwest region of Mexico, specifically in Durango city, elevated concentrations of fluoride have been reported in drinking water, affecting millions of residents. A total of 63 groundwater wells, representing 86% of the city’s total wells, were analyzed, revealing fluoride concentrations ranging from 2.22 to 7.23 ppm. The northern part of the city presented the highest fluoride levels, with a mean concentration of 5.00 (± 2.66 ppm SD) [7]. In 2017, a study in the capital of Durango State examined 308 adolescents from different socioeconomic groups, all of whom presented with dental fluorosis. Using the Thylstrup‒Fejerskov index (TFI) according to WHO guidelines, researchers recorded the highest TFI score per individual. The results revealed that 22.7% of the patients had mild fluorosis (TFI 1–3), 72.1% had moderate (TFI 4–6), and 5.2% had severe (TFI 7–8) [8].

Other factors associated with dental fluorosis include genetic and environmental factors. Animal studies have shown that susceptibility or resistance to dental fluorosis is based on genetic factors. Moreover, genes associated with variations in the predisposition to fluorosis have been identified in individuals from a community that shares the same environmental exposure [9]. Multiple genetic epidemiological studies have presented results on the associations between polymorphisms present in certain genes and susceptibility to different degrees of severity of dental fluorosis in people from the same community with the same environmental exposure [10].

The *DLX* genes are part of the homeobox gene family and were first discovered through mutations in *Drosophila melanogaster* [11]. Homeobox genes primarily encode transcription factors that modulate gene expression through specific DNA binding via the homeodomain and interactions with protein cofactors. These transcription factors are critically involved in the morphogenesis of craniofacial structures, such as the anterior brain, basal telencephalon, diencephalon, prosencephalon, sensory organs, and branchial arches [12–14]. Multiple studies have revealed an association between *DLX3* and the development of dental organs, as well as its relation to alterations therein. Dong et al. [15] analyzed a family of Australian origin with hypoplastic-hypomaturation amelogenesis imperfecta with taurodontism (AIHHT) across three generations, comprising 21 members, 11 of whom had the AIHHT phenotype. They reported an association of a two-nucleotide deletion in *DLX3*, finding that the mutation in exon 3 is associated with AIHHT pathogenesis [15]. A 2015 study by Zhichun Zhang et al., using neonatal murine models, investigated the regulatory role of DLX3 in enamel matrix protein expression. Immunohistochemical analysis revealed strong DLX3 immunostaining in ameloblasts during the secretory stage, with markedly lower expression during the presecretory and maturation stages [16]. Fabiano Jeremias et al. in 2016 [17] focused on investigating genes potentially involved in the development of molar-incisor hypomineralization (HMI), evaluating a total of 391 people and collecting samples of oral epithelial cells. They evaluated 63 SNPs of 21 candidate genes, including *DLX3* rs 2,278,163. This study divided the study population as follows: 165 did not present HMI, 96 had an unknown HMI diagnosis, and 130 had severe HMI. They reported that *DLX3* rs 2,278,163 is a risk factor for the HMI phenotype (OR = 2.8, 1.26–6.44) [17]. Other genes not directly related to enamel formation, such as ESR genes, have also been associated with DF. Estrogen receptors (ERs) have two main subtypes, ERα and ERβ, each encoded by a separate gene (ESR1 and ESR2, respectively). ESR1 and ESR2 are expressed during odontogenesis and may be involved in developmental defects in enamel [18]. These receptors are primarily nuclear proteins that mediate the effects of estrogen by binding to DNA and regulating gene transcription. ESR2 does not seem to be associated with DF, but there is enough evidence to suggest that ESR1 is associated with DF [19, 20].

Chronic exposure to elevated fluoride concentrations remains a significant public health concern in several regions of Mexico, specially in Durango. In recent years, water scarcity has forced many cities to extract water from deeper aquifer levels, a practice that often increases the concentration of fluoride and other elements in drinking water. Pregnant women are a critical group to study because they experience fluoride exposure both directly through the environment and indirectly through physiological changes that may influence fetal development, creating a scenario of “double exposure” with potential long-term consequences. Importantly, the correlation between fluoride levels in water and the clinical severity of dental fluorosis, as measured by the Thylstrup–Fejerskov Index (TFI), is not always linear, suggesting that genetic factors may play a key role in modulating the phenotype. Understanding this interaction is particularly relevant in chronically exposed populations, where inter-individual variability cannot be explained by exposure levels alone. Therefore, this study aimed to investigate whether ESR1 rs12154178 and ESR2 rs1256049 polymorphisms are associated with the severity of dental fluorosis in pregnant women from Durango, Mexico.

## Methods

### Study design and sample collection

This cross-sectional case–control study included 79 women residing in Durango, Mexico, a region with elevated fluoride concentrations in drinking water, sample was calculated using G*Power (v3.1). Dental fluorosis was classified using the Thylstrup–Fejerskov Index (TFI). No participants without fluorosis (TFI = 0) were identified in the cohort, indicating that all individuals presented some degree of fluorosis. A small number of participants with severe fluorosis (TFI ≥ 7) were identified; however, these cases (*n* < 5) were excluded from the genetic association analysis because their limited representation precluded meaningful statistical inference. We considered pooling severe cases with less severe categories, but clinical distinctions between moderate and severe fluorosis—particularly enamel surface loss, porosity, and functional compromise—supported a conservative phenotype definition. Accordingly, two phenotypic groups were analyzed: TFI 1–3 and TFI 4–6. This classification ensured phenotypic homogeneity within groups and adequate power for the comparisons performed. Data were collected at a single time point and analyzed using descriptive and analytical statistics. All women were born and raised in the city of Durango and agreed to participate by signing an informed consent form between 2021 and 2023.

The inclusion of pregnant women was based on the broader objective of a larger, longitudinal project investigating the potential effects of chronic fluoride and arsenic exposure on maternal and child health. Studying this population enabled consistent timing of biological sample collection and facilitated the future follow-up of mother–child pairs, thereby strengthening both environmental and genetic analyses.

The objective was to evaluate the association between specific genetic polymorphisms (DLX3, ESR1, and ESR2) and the severity of dental fluorosis, and to examine environmental fluoride exposure by analyzing tap water, bottled drinking water, urine, and blood samples, and analyze phenotypic variation.

Women undergoing orthodontic treatment or any dental procedure that could obstruct the diagnosis of dental fluorosis were excluded. Participants were also removed from the study if they voluntarily withdrew or if their biological samples were found to be damaged, contaminated, or insufficient for analysis.

This study focused exclusively on pregnant women as part of a larger, ongoing longitudinal research initiative designed to investigate the impact of environmental exposures—particularly fluoride and arsenic—on maternal and child health.

Tap water samples were taken directly from the kitchen faucet of each participant’s household. These samples represent the primary source of potential environmental exposure through household use (cooking, brushing teeth).

Drinking water was defined as bottled water used by participants for direct consumption. All participants reported drinking only bottled water, which was purchased from local private suppliers. No participants reported consuming tap water. Urine samples were collected using sterile, fluoride-free polypropylene containers provided during the home visit. Participants were instructed to provide a first-morning midstream urine sample, when possible, to reduce variability due to diurnal excretion patterns. Samples were immediately placed in cool storage and later transported to the laboratory under cold chain conditions for fluoride quantification.

Blood samples were collected via venipuncture into EDTA-coated tubes. Approximately 5 mL of whole blood was drawn from each participant. Samples were centrifuged to separate plasma and stored at − 80 °C until analysis. Blood was used for genotyping and other biochemical assessments related to the broader study objectives.

While current fluoride exposure was measured through household water sampling, historical childhood exposure could not be directly assessed. All participants, however, were lifelong residents of Durango, a region with long-term endemic fluoride exposure.

### Clinical evaluation

The clinical evaluation was conducted according to the principles established by the World Health Organization in 1987; a No. 5 intraoral mirror was used under natural light, and dental plaque was removed with gauze. The evaluation was conducted in the field in quadrants, starting with the upper right molars and ending with the lower left molars. The diagnosis of dental fluorosis was carried out according to the Thylstrup and Fejerskov indices, and intraoral photographs were taken for case documentation and by a calibrated and professional dentist with a kappa coefficient of 0.92 at the person and tooth levels. The participants were classified as follows: sound enamel (TFI = 0), mild (TFI = 1–3) fluorosis, but no loss of the enamel surface; moderate (TFI = 4–6) loss of the enamel surface with small craters; and severe (TFI = 7–9, significant loss of the enamel surface, up to more than 50%), as suggested by Zepeda et al. [6]

### Fluoride quantification

The urine analyzed for this study was collected throughout the day during the first trimester of pregnancy, frozen at −80 °C along with water, and subsequently analyzed. The quantification of fluoride in water and urine was conducted via potentiometry with an Orion™ Dual Star™ pH/ISE meter (Thermo Scientific™, 2115000) and an Orion™ fluoride ion selective electrode (Thermo Scientific™, 9609BNWP), following the manufacturer’s established protocol. All the water and urine samples were analyzed in triplicate. A standard curve using concentrations of 1, 3, 5, 7, 10 and 20 ppm, which was also run in triplicate, was generated prior to each determination.

### DNA Extraction and genotyping

Peripheral blood samples were processed on the basis of the DNA extraction protocol by Mobarakeh Iranpur from 2010 [21]. The quantification and purity of the DNA samples were evaluated via spectrophotometry via a NanoDrop Lite (Thermo Scientific™). Genotyping was performed via TaqMan probes for rs2278163, rs12154178, and rs1256049 in a 48-well real-time PCR Kit ONE system (Applied Biosystems) under the following conditions: stage (1) 60 °C for 30 s, stage (2) 95 °C for 10 min, stage (3) 92 °C for 15 s, stage (4) 60 °C in 1 min, stage (5) 40 cycles of stages 2, 3, and 4, and stage (6) 60 °C for 30 s.

### Statistical analysis

Rstudio 2025.09.0–387.0 was utilized to perform descriptive and inferential statistical analyses. Measures of central tendency, dispersion, and frequency were computed. Group comparisons were conducted using t tests for normally distributed variables and Mann–Whitney U tests for non-parametric data. Pearson correlation coefficients were calculated to assess the relationships between continuous variables.

To evaluate genetic associations, logistic regression models were applied to estimate odds ratios (ORs) and 95% confidence intervals (CIs) under additive, dominant, and recessive inheritance models. The outcome variable was fluorosis severity, categorized as mild (TFI 1–4) versus moderate (TFI 5–6). Severe cases (TFI ≥ 7) were excluded from this analysis due to their small number. Each genotype was tested for association with fluorosis severity using binary logistic regression, and Hardy–Weinberg equilibrium was verified for each SNP using chi-square tests.

## Results

**Fig. 1:**
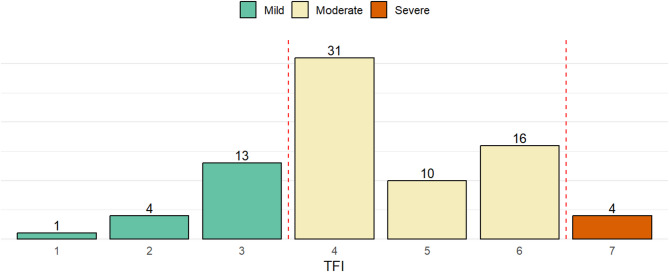
Distribution of thylstrup–fejerskov index (TFI) scores and fluorosis severity categories

A total of 79 individuals were categorized based on their highest TFI score, sociodemographic and clinical data is shown in Table 1. Most participants fell within the moderate fluorosis category (TFI 4–6), with TFI 4 being the most frequent score (*n* = 31), followed by TFI 6 (*n* = 16) and TFI 5 (*n* = 10). The mild fluorosis group (TFI 1–3) included 18 individuals, with TFI 3 being the most common (*n* = 13). Only 4 participants were classified as having severe fluorosis (TFI ≥ 7). The red dashed lines indicate the thresholds between severity groups, as defined by Zepeda et al. [6] (Fig. 1).

**Table 1:** Sociodemographic and clinical profile of participants

Variable	Categories/Statistics	*n* (%)/Mean ± SD/Range
Age (years)	Mean ± SD	27.1 ± 6.0
Marital status	Single Married/Union Other	23.7% 44.1% 30.1%
Education	Secondary Highschool University	2.2% 40.9% 55.9%
Weight (kg)	Mean ± SD	64.6 ± 15.6
Height (cm)	Mean ± SD	161.4 ± 7.1
BMI (kg/m²)	Mean ± SD	24.6 ± 4.7
Number of pregnancies	Mean ± SD	2.0 ± 1.1
Gestational weeks at sample	Mean ± SD	14.6 ± 5.7

Fluoride was assessed in three matrices: tap water (water directly collected from household plumbing), drinking water (typically bottled or filtered water, distinct from tap water in most cases), and urine (measured from a full-day urine collection to estimate systemic fluoride exposure). The mean fluoride concentrations were 5.38 ± 2.73 ppm in tap water, 2.32 ± 2.78 ppm in drinking water, and 4.79 ± 4.60 ppm in urine. The participants were stratified based on fluoride concentration in their drinking water, with a cutoff point of 1.5 ppm.

The participants consuming water with ≥ 1.5 ppm fluoride presented significantly higher urinary fluoride levels (6.06 ppm [3.37–12.04]) than did those consuming < 1.5 ppm fluoride (2.37 ppm [1.14–4.32]; *p* = 0.0001). In terms of hematological parameters, the mean corpuscular volume (MCV) was significantly lower in the high-fluoride group (91.00 [88–92.00]) than in the low-fluoride group (94.35 [89.83–98.73]; *p* = 0.0103), whereas monocyte counts were significantly higher (6.90 [5.5–9.05] vs. 5.40 [4.8–8.85]; *p* = 0.0494). No significant differences were observed in hemoglobin levels, erythrocyte counts, hematocrit, BMI, age, or platelet parameters between the exposure groups (Table 2).

**Table 2:** Comparison of hematological and clinical parameters by fluoride exposure in drinking water

Variable	< 1.5 ppm F	≥ 1.5 ppm F	*p*_value
Erythrocyte	4.21 (3.9625–4.51)	4.50 (4.18–4.60)	0.0736
F ppm Urine	2.37 (1.14–4.32)	6.06 (3.365–12.04)	0.0001*
Hematocrit	39.80 (37.5–41.92)	40.00 (38–42.00)	0.8875
IMC	26.40 (22.925–30.43)	24.80 (21.3–29.05)	0.3149
Leucocytes	7800.00 (6875–8950.00)	8800.00 (7300–9500.00)	0.2410
Lymphocyte	22.60 (18.625–28.25)	25.50 (21.45–28.93)	0.5511
Mean Corpuscular Volume	94.35 (89.825–98.73)	91.00 (88–92.00)	0.0103*
Mean Platelet Volume	8.15 (7.6–8.65)	8.05 (7.5–9.12)	0.8918
Monocytes	5.40 (4.8–8.85)	6.90 (5.5–9.05)	0.0494*
Platelet	213,500(184250–254000)	234,500 (207750–246000)	0.5167
Red Cell Distribution Width	11.80 (11.45–12.70)	12.15 (11.75–12.85)	0.4973
Age	26.64 ± 6.13	25.86 ± 4.93	0.5561
Hemoglobin	12.65 ± 1.14	12.55 ± 1.33	0.7961
Mean Corpuscular Hb	29.52 ± 2.66	28.26 ± 2.72	0.1127

**Fig. 2:**
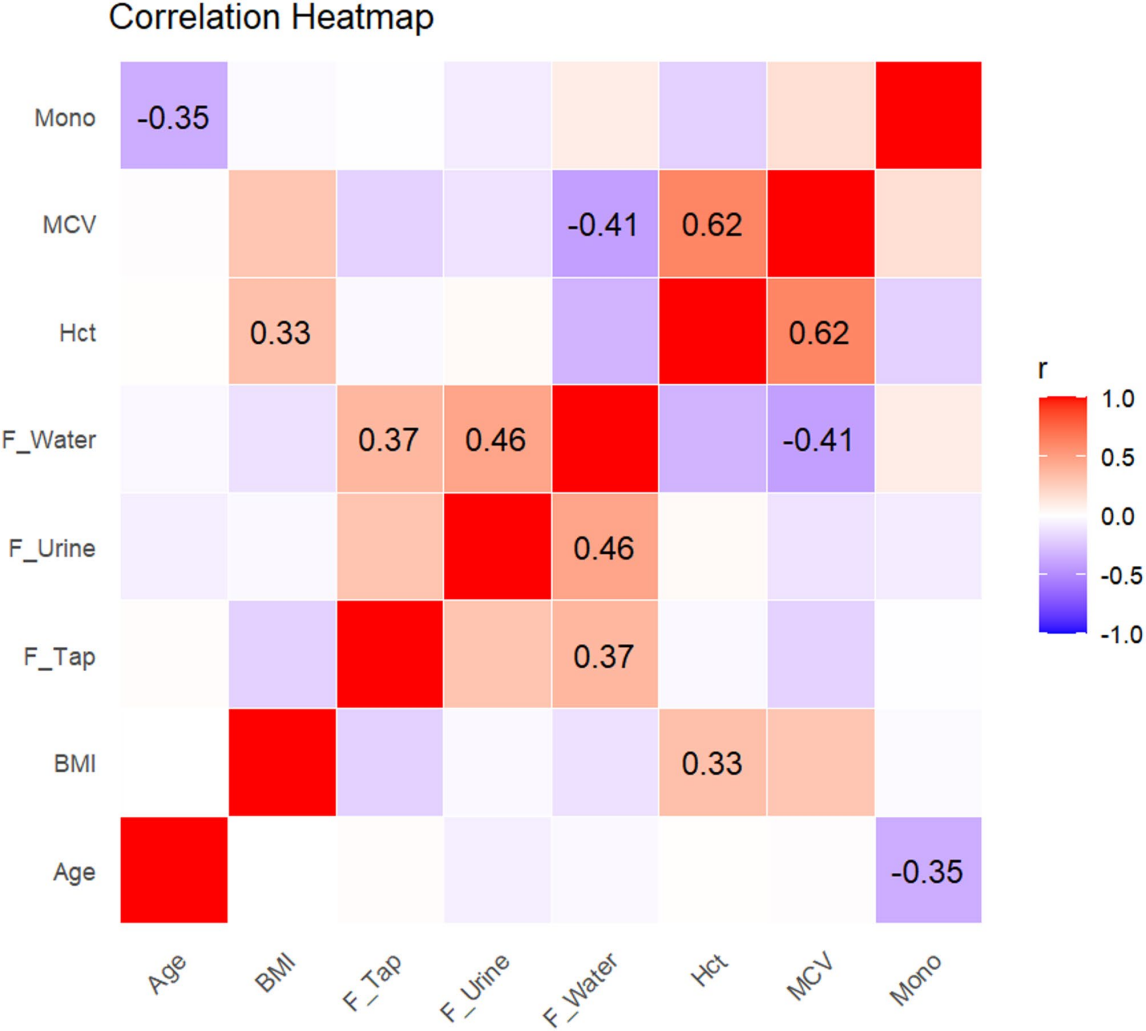
Significant correlations between fluoride levels and clinical parameters

A correlation analysis revealed several statistically significant associations. The fluoride concentration in drinking water was positively correlated with the urinary fluoride level (*r* = 0.46, *p* = 0.0039) and with the fluoride level in tap water (*r* = 0.37, *p* = 0.0245). Conversely, it was negatively correlated with the mean corpuscular volume (MCV) (*r* = − 0.41, *p* = 0.0119). Age was inversely correlated with monocyte count (*r* = − 0.35, *p* = 0.0326), and hematocrit was positively correlated with body mass index (BMI) (*r* = 0.33, *p* = 0.0464) (Table 3). These relationships are visually represented in the correlation heatmap (Fig. 2), where the strength and direction of the correlations are depicted through color intensity, with significant values labeled.

**Table 3:** Correlation analysis between fluoride exposure and clinical variables

Variable 1	Variable 2	*r*	*p*
F in Drinking Water	F in Urine	0.46	0.00392
F in Drinking Water	Mean Corpuscular Volume	−0.41	0.01190
F Drinking Water	F Tap Water	0.37	0.02450
Age	Monocyte	−0.35	0.03260
Hematocrit	Body Mass Index	0.33	0.04640

Allele frequencies were evaluated for three single nucleotide polymorphisms (SNPs): rs2278163 (DLX3), rs12154178 (ESR1), and rs1256049 (ESR2). For rs2278163, the G allele was more common (70.8%) than the A allele was (29.2%), based on a total of 142 alleles. For rs12154178, the C allele represented 57.0%, and the A allele represented 43.0% of 146 alleles. For rs1256049, the C allele was highly predominant (97.0%), whereas the T allele was observed in only 3.0% of the 146 total alleles (Table 4).

**Table 4:** Allele frequency distributions of the DLX3, ESR1, and ESR2 SNPs

SNP	Gene	Allele	*N*	Percentage
rs2278163	DLX3	A	42	29.2
		G	102	70.8
		Total	144	100.0
rs12154178	ESR1	A	63	43.0
		C	83	57.0
		Total	146	100.0
rs1256049	ESR2	T	4	3.0
		C	142	97.0
		Total	146	100.0

For rs2278163 (DLX3), the G/G genotype was most common (52.7%), followed by the A/G (36.1%) and A/A (11.1%) genotypes, among the 72 individuals. For rs12154178 (ESR1), the heterozygous C/A genotype predominated (45.0%), with C/C and A/A observed in 34.0% and 21.0% of participants, respectively (*n* = 73). For rs1256049 (ESR2), the C/C genotype was found in 95.0% of the participants, the C/T genotype was found in 5.0%, and no individuals carried the T/T genotype (*n* = 73) (Table 5).

**Table 5:** Genotype frequency distributions for the DLX3, ESR1, and ESR2 SNPs

SNP	Gene	Genotype	*N*	Percentage
rs2278163	DLX3	A/A	8	11.1
		A/G	26	36.1
		G/G	38	52.7
		Total	72	100.0
rs12154178	ESR1	A/A	15	21.0
		C/A	33	45.0
		C/C	25	34.0
		Total	73	100.0
rs1256049	ESR2	C/T	4	5.0
		C/C	69	95.0
		T/T	0	0.0
		Total	73	100.0

Under the codominant model, individuals with the A/G genotype of DLX3 rs2278163 were significantly less likely to present moderate fluorosis compared to those with mild fluorosis (OR = 0.00, 95% CI: 0.00–NA; *p* = 0.028). In the recessive model, the G/G genotype was associated with reduced odds of moderate fluorosis relative to carriers of at least one A allele (A/A + A/G), with an odds ratio of 0.25 (95% CI: 0.07–0.88; *p* = 0.022). The dominant model also suggested a protective effect for the combined A/G + G/G genotypes compared with A/A, although this association did not reach statistical significance (*p* = 0.052) (Table 6).

**Table 6:** Genetic association models for DLX3 (rs2278163)

Model	Genotype	Mild	Moderate	OR_CI	*p*_value
Codominant	A/A	0	6		-
	A/G	4	21	0.00 (0.00–NA)	0.028
	G/G	13	22	0.00 (0.00–NA)	-
Dominant	A/A	0	6		-
	A/G + G/G	17	43	0.00 (0.00–NA)	0.052
Recessive	A/A + A/G	4	27		-
	G/G	13	22	0.25 (0.07–0.88)	0.022
Overdominant	A/A + G/G	13	28		-
	A/G	4	21	2.44 (0.69–8.55)	0.15

The overdominant model for ESR1 rs12154178 showed a statistically significant association: individuals with the C/A genotype had lower odds of presenting moderate fluorosis than those with homozygous genotypes (C/C + A/A) did (OR = 0.31, 95% CI: 0.10–0.95; *p* = 0.034). A similar trend was observed under the codominant model, where the C/A genotype was associated with lower odds of moderate fluorosis (OR = 0.24, 95% CI: 0.06–0.97), although the result approached but did not reach conventional significance (*p* = 0.085). The dominant model also suggested a protective association for carriers of at least one A allele (C/A + A/A) compared with C/C (OR = 0.30, 95% CI: 0.08–1.16; *p* = 0.059) (Table 7).

**Table 7:** Genetic association models for ESR1 (rs12154178)

Model	Genotype	Mild	Moderate	OR_CI	*p*_value
Codominant	C/C	3	22	1.00	-
	C/A	12	21	0.24 (0.06–0.97)	0.085
	A/A	3	12	0.55 (0.09–3.13)	-
Dominant	C/C	3	22	1.00	-
	C/A + A/A	15	33	0.30 (0.08–1.16)	0.059
Recessive	C/C + C/A	15	43	1.00	-
	A/A	3	12	1.40 (0.35–5.63)	0.63
Overdominant	C/C + A/A	6	34	1.00	-
	C/A	12	21	0.31 (0.10–0.95)	0.034
Log-additive	---	---	---	0.71 (0.34–1.48)	0.36

For ESR2 rs1256049, no statistically significant association with fluorosis severity was observed. Although the C/T genotype showed lower odds ratio than the C/C genotype did (OR = 0.30, 95% CI: 0.04–2.32), the difference was not significant (*p* = 0.26) (Table 8).

**Table 8:** Genetic association models for ESR2 (rs1256049)

Model	Genotype	Mild	Moderate	OR_CI	*p*_value
---	C/C	16 (88.9%)	53 (96.4%)	1.00	-
---	C/T	2 (11.1%)	2 (3.6%)	0.30 (0.04–2.32)	0.26

## Discussion

In this study, fluoride exposure was assessed via three matrices: tap water (collected directly from household plumbing), drinking water (typically bottled or filtered and distinct from tap water), and urinary fluoride, which was obtained through 24-hour collection to estimate systemic fluoride intake. The participants were stratified by their drinking water fluoride concentration using a 1.5 ppm threshold, in accordance with the World Health Organization (WHO) and Mexican Official Norms (NOM) guidelines [22]. In contrast, no threshold was applied to urinary fluoride, as neither the WHO nor the NOM currently defines reference values for this biomarker.

The mean concentrations of fluoride found in tap and drinking water in this study were higher than those reported in earlier evaluations of groundwater wells in Durango [7]. This may indicate increased exposure over time due to changes in water sourcing or treatment, which aligns with the clinical findings: no participant presented with a TFI of 0, meaning that 100% of the sample exhibited some degree of dental fluorosis. Nevertheless, the number of individuals classified with severe fluorosis (TFI ≥ 7) was low. Among the 79 participants, 100% consumed bottled water for drinking. Despite this, 100% presented with fluorosis (TFI ≥ 1), reflecting likely exposure during earlier life stages.

This imbalance—universal presence of fluorosis but limited progression to severe forms—may be partially explained by genetic factors that modulate individual susceptibility to fluoride. Indeed, our results revealed polymorphisms in DLX3 and ESR1 that were more common in individuals with mild fluorosis, suggesting that these SNPs may act as protective factors, reducing the likelihood of severe enamel alteration even in the context of elevated fluoride exposure. The association between fluoride in drinking water and urinary fluoride confirmed systemic intake, whereas inverse correlations with the mean corpuscular volume (MCV) and monocyte count raise the possibility of additional physiological effects beyond the dentition. These findings support previous research highlighting the systemic impact of chronic fluoride intake and suggest the need for a more comprehensive evaluation of health outcomes in exposed populations [8].

Genetic comparisons focused on individuals with mild and moderate fluorosis only, as the severe group was excluded from association testing because of an insufficient sample size for valid statistical inference. Within this subset, polymorphisms in DLX3 (rs2278163) and ESR1 (rs12154178) are associated with fluorosis severity, indicating a role in modulating the phenotypic expression of enamel defects. These genes have been previously implicated in enamel development. DLX3 encodes a transcription factor critical for craniofacial and dental morphogenesis, and previous studies have linked mutations in DLX3 to conditions such as amelogenesis imperfecta and molar-incisor hyponmineralization [16]. ESR1 encodes estrogen receptor alpha, which is involved in hormonal regulation during tooth development [20]. The presence of specific alleles may alter the responsiveness of ameloblasts to environmental stressors such as fluoride, ultimately influencing enamel integrity.

In contrast, ESR2 (rs1256049) showed no significant association with fluorosis severity, which is consistent with earlier findings that suggest a more limited role of this gene in enamel formation. The divergence in impact between ESR1 and ESR2 highlights the specificity of gene–environment interactions in dental tissue development.

Together, these findings reinforce the concept that dental fluorosis is not solely a result of environmental exposure but is shaped by the interplay between fluoride intake and host genetic susceptibility. In regions such as Durango, where exposure levels remain high, identifying protective or risk-associated genotypes could inform individual risk assessments, early intervention strategies, and public health planning.

## Conclusions

Our findings support a multifactorial model for dental fluorosis, where both environmental fluoride exposure and the host genetic background influence disease expression. The universal presence of fluorosis in this cohort, coupled with the limited number of severe cases and the identification of potentially protective genotypes in DLX3 and ESR1, suggests that genetic variation plays a meaningful role in modulating individual susceptibility. These results underscore the need to integrate molecular analysis into fluorosis risk assessment, particularly in high-exposure regions. This study has several limitations. First, all participants were pregnant women, and physiological changes during pregnancy may influence fluoride metabolism and the expression of fluorosis. Second, the sample size was relatively small (*N* = 79), which may have limited statistical power to detect modest genetic effects. Finally, because dental fluorosis reflects fluoride exposure during enamel formation, the ideal time for assessing exposure would have been during the participants’ childhood; thus, retrospective reconstruction of exposure remains a challenge. These factors should be considered when interpreting the findings and their generalizability.

## Data Availability

The datasets used and/or analyzed during the current study are available from the corresponding author upon reasonable request. The genetic variant data related to this study have been registered under BioProject accession PRJNA1291116 at the NCBI. The full variant dataset will be made publicly available upon completion of processing in dbSNP.
